# Associations Between Cognitive Impairment, Depressive Symptoms, and Work Productivity Loss in Patients With Bipolar Disorder: A Cross‐Sectional Analysis

**DOI:** 10.1002/npr2.70012

**Published:** 2025-03-20

**Authors:** Yoshikazu Takaesu, Ayano Shiroma, Tadashi Nosaka, Hidenori Maruyama

**Affiliations:** ^1^ Department of Neuropsychiatry, Graduate School of Medicine University of the Ryukyus Okinawa Japan; ^2^ Medical Affairs Sumitomo Pharma Co. Ltd. Tokyo Japan

**Keywords:** bipolar disorder, cognitive impairment, depression, presenteeism, quality of life

## Abstract

**Aim:**

To evaluate the relationship between cognitive impairment and work productivity loss in patients with bipolar disorder.

**Methods:**

We enrolled outpatients with bipolar disorder aged 18–59 years undergoing treatment and actively employed or on sick leave. Baseline demographic, medical resource use, and employment data were collected. We evaluated work productivity, cognitive impairment, quality of life (QOL), depressive symptoms (defined as a Patient Health Questionnaire‐9 [PHQ‐9] score of ≥ 10), and sleep disturbance. This interim analysis examined correlations among baseline symptom scores and correlations of each symptom score with work productivity loss and QOL.

**Results:**

Among 211 participants, cognitive impairment was moderately correlated with depressive symptoms (*r* = 0.595) and insomnia (*r* = 0.481), and depressive symptoms and insomnia were highly correlated (*r* = 0.719) (all *p* < 0.001). Work productivity loss (presenteeism) was moderately correlated with cognitive impairment (*r* = 0.474), depression (*r* = 0.577), and insomnia (*r* = 0.547) (all *p* < 0.001). Depression had the strongest influence on presenteeism (multiple regression analysis, regression coefficient: 22.98; *p* < 0.001). Among participants without severe depressive symptoms (PHQ‐9 ≤ 19), cognitive impairment (13.91, *p* = 0.007) and insomnia (13.80, *p* = 0.016) strongly affected presenteeism. Among participants without moderately severe or severe depressive symptoms (PHQ‐9 ≤ 14), insomnia affected presenteeism (23.14, *p* = 0.011). QOL was moderately negatively associated with cognitive impairment (*r* = −0.653), depression (*r* = −0.699), and insomnia (*r* = −0.559) (all *p* < 0.001). In multiple regression analysis, cognitive impairment (−0.12, *p* < 0.001), depression (−0.12, *p* = 0.010), and insomnia (−0.16, *p* < 0.001) were significantly associated with QOL.

**Conclusions:**

Treatment should focus on improving the core symptoms of bipolar disorder, insomnia, and cognitive impairment.

**Trial Registration:**

UMIN Clinical Trials Registry (UMIN000051519)

## Abstract 2

This interim, cross‐sectional analysis to evaluate the relationship between cognitive impairment and work productivity loss in patients with bipolar disorder examined correlations among baseline symptom scores and correlations between each symptom score and work productivity loss (presenteeism) and quality of life. Cognitive impairment, depression, and insomnia were associated with both presenteeism and quality of life. The study findings suggest that treatment should focus on improving the core symptoms of bipolar disorder as well as insomnia and cognitive impairment.

## Graphical abstract

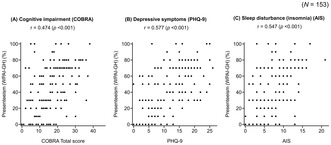

## 1 Introduction

Bipolar disorder is a type of mood disorder in which patients experience recurrent manic and depressive episodes [1]. A remission period is defined as minimal or no episodes of either depression or mania for at least a week [2]. The disorder is classified into two types: bipolar I, which is characterized by intense mania that may have been preceded or followed by a major depressive or hypomanic episode (not required for the diagnosis), and bipolar II, which is characterized by both hypomania and major depressive episodes with no manic episodes [3]. An international survey reported lifetime global prevalence rates of 0.6% and 0.4% for bipolar I and bipolar II, respectively [4]. In Japan, the combined lifetime prevalence of types I and II is approximately 0.2%–0.6% [5, 6, 7]. One prospective study found that over a 13‐year observation period, patients with bipolar I experienced depressive symptoms in 31.9% of weeks during the total observation period (67.7% of weeks excluding the remission phase) and manic/hypomanic symptoms in 8.9% of weeks [8]. Patients with bipolar II experienced depressive symptoms in 50.3% of weeks during the total observation period (93.3% excluding the remission phase) and hypomanic symptoms in 1.3% of weeks [9]. These findings indicate that patients with bipolar disorder primarily experience depressive symptoms.

Patients with bipolar depression exhibit core symptoms such as loss of interest, pleasure, and satisfaction, as well as symptoms of anxiety [10], sleep disturbances [11, 12], and cognitive impairment [13, 14, 15, 16]. Bipolar disorder is associated with cognitive deficits and poor occupational functioning [17]. Furthermore, cognitive impairment in euthymic bipolar disorder is associated with reduced social functioning [15, 18, 19, 20] and quality of life (QOL) [21, 22, 23]. A previous cross‐sectional study reported that patients with bipolar disorder had lower work productivity than adults who had never experienced bipolar disorder, schizophrenia, or major depressive disorder [7]. Another cross‐sectional study that assessed cognitive impairment in general adult workers in Japan using the Cognitive Complaints in Bipolar Disorder Rating Assessment (COBRA) found that cognitive complaints were associated with reduced work productivity [24]. To our knowledge, no studies have clarified the relationship between cognitive impairment and work productivity loss in patients with bipolar disorder, and no longitudinal studies have been conducted.

We are currently conducting a longitudinal study to evaluate the relationship between changes in cognitive function and changes in work productivity in patients with bipolar disorder aged 18–59 years who are attending a medical institution and working (employed or on sick leave) during the 48‐week observation period. Depression, sleep disturbance, QOL, use of medical resources, and employment status are also being evaluated. Here, we report a cross‐sectional analysis of the results obtained at baseline from this ongoing longitudinal study.

## 2 Methods

### 2.1 Study Design and Participants

This is a prospective cohort questionnaire study of patients with bipolar disorder that is being conducted in Japan. The study was initiated on July 7, 2023 following approval by the head of the University of the Ryukyus. This planned interim analysis was conducted after the completion of participant enrollment.

The study includes patients with bipolar disorder who were registered in the QLife web system (QLife Inc., Tokyo, Japan) and who were being treated for bipolar disorder at a medical institution at the time of the first questionnaire assessment. Participants were required to be ≥ 18 years of age and ≤ 59 years of age at the time informed consent (via the web system). Additionally, participants had to be employed or on sick leave and able to complete the web questionnaire at the time of evaluation. Participants who did not plan to return to work or find employment in the next 12 months, were hospitalized at baseline, or were diagnosed with dementia at baseline were excluded.

This study adhered to the principles set forth in the Declaration of Helsinki, the Ethical Guidelines for Life Sciences and Medical Research Involving Human Subjects, and all applicable laws and regulations. The study protocol was approved by the University of the Ryukyus Ethics Review Committee for Life Science and Medical Research Involving Human Subjects. Participants provided electronic informed consent prior to initiating the study questionnaire.

### 2.2 Questionnaire Implementation

Participants were registered by the questionnaire administrator (QLife Inc.). All questionnaire responses were recorded in the QLife web system. Questionnaires were administered online at the time of study enrollment (baseline) and participants received a link to the study questionnaire via email or SMS, using the email address or phone number registered during the baseline survey.

### 2.3 Questionnaire Items

The baseline questionnaire recorded participant background information, including name, email address, and phone number, which were only used for communications from the questionnaire administrator. The baseline (Week 0) questionnaires asked participants about who they were living with, their employment status, whether they had public health insurance, and their eating, sleeping, and exercise habits. All questionnaires included the following self‐assessments. Cognitive impairment was assessed using the COBRA [25, 26], from which a total score for each was calculated. The Work Productivity and Activity Impairment Questionnaire General Health (WPAI‐GH) [27, 28] was used to evaluate presenteeism, absenteeism, overall work impairment, and activity impairment. Medical resource use and employment status were assessed from the number of hospital visits, hospitalizations, and days in hospital, as well as hospitalization rate and rates of absence from work and return to work. QOL was assessed using the Health Utilities Index Mark 3 (HUI3) [29, 30], from which a total utility value and utility values for each of the eight items (vision, hearing, speech, ambulation, dexterity, emotion, cognition, and pain) were calculated (scores of 1.00 indicate perfect health). Depressive symptom severity was assessed using the Patient Health Questionnaire‐9 (PHQ‐9) [31, 32], from which the total depressive symptom severity score was calculated. Sleep disturbance was assessed using the Epworth Sleepiness Scale (ESS) [33, 34] and the Athens Insomnia Scale (AIS) [35, 36], from which a total score for each was calculated.

### 2.4 Study Endpoints

This interim analysis reports baseline participant characteristics, correlations among baseline symptom scores (cognitive impairment, COBRA; depressive symptoms, PHQ‐9; insomnia, AIS), correlations between each symptom score and work productivity loss (WPAI‐GH [Presenteeism, Absenteeism, Overall work impairment, Activity impairment]) using single regression analysis and multiple regression analysis, and correlations between each symptom score and QOL (HUI3) using single regression analysis and multiple regression analysis.

### 2.5 Statistical Analysis

All participants who met the eligibility criteria and whose questionnaire responses were valid were included in the analysis population. Summary statistics were used to report categorical (*n* [%]) and continuous (mean [standard deviation, SD]) variables.

For each assessment measure (COBRA, PHQ‐9, ESS, and AIS), participants were assigned to a comparison group per their baseline score (absence vs. presence of each variable). The cutoff scores for absence or presence were as follows: COBRA ≤ 14 versus ≥ 15 (cognitive impairment) [37], PHQ‐9 ≤ 9 versus ≥ 10 (depressive symptoms) [7, 32, 38], ESS ≤ 10 versus ≥ 11 (somnolence) [34], AIS ≤ 9 versus ≥ 10 (insomnia) [35]. Regarding other cutoff scores, PHQ‐9 ≥ 15 was used to define moderately severe or severe depression (PHQ‐9 ≤ 14, moderate or less [mild or none/minimal] depressive symptoms) and PHQ‐9 ≥ 20 was used to define severe depression (PHQ‐9 ≤ 19, moderately severe or less [moderate, mild, or none/minimal] depressive symptoms).

In this interim analysis, correlations between symptoms (cognitive impairment and depressive symptoms, cognitive impairment and insomnia, and depressive symptoms and insomnia) and between work productivity (presenteeism) or QOL (HUI3) and symptoms (cognitive impairment, depressive symptoms, and insomnia) at baseline (Week 0) were determined by creating scatter plots and calculating Pearson's correlation coefficients. Additionally, exploratory single and multiple regression analyses were conducted with work productivity (WPAI‐GH) (in the total population, the population with baseline PHQ‐9 ≤ 19 [without severe depressive symptoms], and the population with baseline PHQ‐9 ≤ 14 [without moderately severe or severe depressive symptoms]) and QOL (HUI3) as the objective variables and baseline participant characteristics and each symptom as explanatory variables. In the multiple regression analysis, explanatory variables were selected based on multiple aspects: results of single regression analysis, clinical perspectives, appropriate number of explanatory variables based on the number of study populations, and avoidance of multicollinearity.

Indirect cost was calculated using the Basic Survey on Wage Structure [39] and the following formula: absenteeism cost = applicable monthly wage by sex and age × absenteeism ratio; presenteeism cost = applicable monthly wage by sex and age × presenteeism ratio; indirect cost = applicable monthly wage by sex, age × overall work impairment ratio (%) (for annual salary × 12 months).

Statistical analyses were performed using SAS version 9.4 software (SAS institute Inc., Cary, NC, USA). All statistical tests were two‐tailed and had a 5% level of significance, with no adjustment for multiple comparisons.

## 3 Results

### 3.1 Participant Characteristics

**FIGURE 1:**
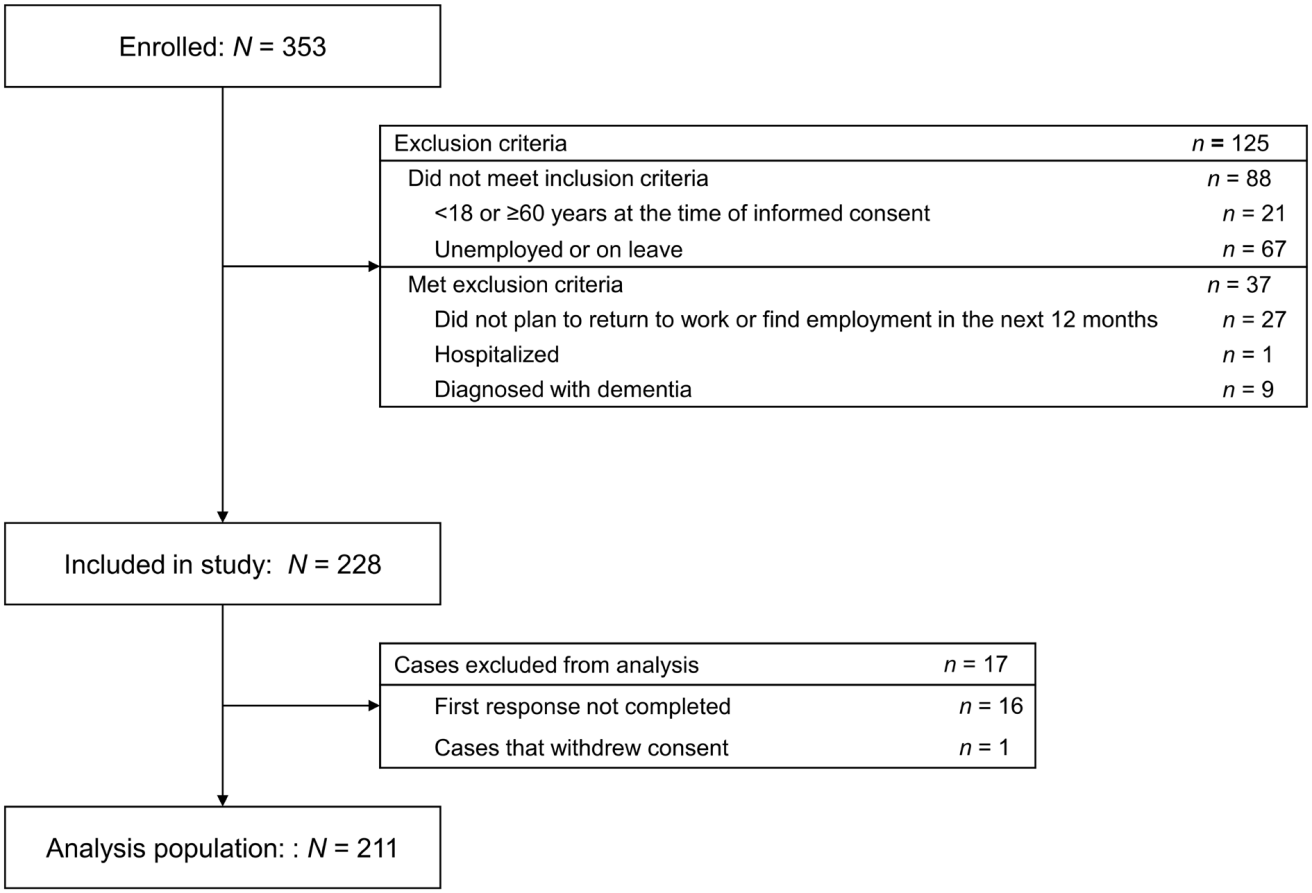
Patient disposition.

In total, 353 participants with bipolar disorder were enrolled in the study and provided electronic informed consent (Figure 1). Of those, 125 participants were ineligible for study participation according to the inclusion and exclusion criteria. There were 228 participants included in the study, 17 of whom were excluded from the analysis (first response not completed, *n* = 16; withdrew consent, *n* = 1). In total, 211 participants were included in the analysis population.

The mean ± SD age of participants was 38.9 ± 10.3 years, 64.9% were female, and 41.2% were working full time and had a mean ± SD current employment duration of 4.6 ± 7.3 years (Table 1). Participants had a mean ± SD duration of bipolar disorder of 7.2 ± 6.8 years, and 40.3% had comorbidities. Cognitive impairment was present (COBRA ≥ 15) in 59.7% of participants; depressive symptoms were present (PHQ‐9 ≥ 10) in 67.8% of participants; the mean WPAI‐GH presenteeism was 45.1%; the mean indirect cost was 1688.0 thousand Japanese yen; and the mean QOL utility value (HUI3) was 0.461.

**TABLE 1:** Participant baseline data.

	Total population (*N* = 211)
Sex	
Male	72 (34.1)
Female	137 (64.9)
No response	2 (0.9)
Age, years	38.9 ± 10.3
Employment status	
Working: full time	87 (41.2)
Working: part time	50 (23.7)
Working: self‐employed	10 (4.7)
Working: continuous employment support	18 (8.5)
Transition support for employment	8 (3.8)
On sick leave	38 (18.0)
Duration of current employment, years	4.6 ± 7.3
Comorbidity	85 (40.3)
Duration of disease, years	7.2 ± 6.8
Age at diagnosis, years	31.7 ± 9.2
Highest level of education	
Did not graduate university	126 (59.7)
University graduate or higher	74 (35.1)
Unknown	11 (5.2)
Married	78 (37.0)
Living with partner (not single)	155 (73.5)
Public health insurance	
Non‐national health insurance	140 (66.4)
Alcohol use	
None	69 (32.7)
≤ once/month	52 (24.6)
2–4 times/month	50 (23.7)
2–3 times/week	15 (7.1)
≥ 4 times/week	25 (11.8)
Smoking history	
Never smoker	121 (57.3)
Current smoker	64 (30.3)
Prior smoker	26 (12.3)
Sleep duration, hours/day	
Weekdays	7.4 ± 1.6
Weekends	8.3 ± 2.1
Eating habits	
Very regular	41 (19.4)
Somewhat regular	72 (34.1)
Slightly irregular	62 (29.4)
Very irregular	36 (17.1)
Exercise habits, *n* = 210^a^, steps/day	4671.0 ± 5849.2
Cognitive impairment (COBRA)	17.6 ± 8.9
Absence (COBRA ≤ 14)	85 (40.3)
Presence (COBRA ≥ 15)	126 (59.7)
Depressive symptoms (PHQ‐9)	13.1 ± 6.6
Absence (PHQ‐9 ≤ 9)	68 (32.2)
Presence (PHQ‐9 ≥ 10)	143 (67.8)
Sleep disturbance (somnolence) (ESS)	9.9 ± 5.6
Absence (ESS ≤ 10)	122 (57.8)
Presence (ESS ≥ 11)	89 (42.2)
Sleep disturbance (insomnia) (AIS)	8.2 ± 4.5
Absence (AIS ≤ 9)	127 (60.2)
Presence (AIS ≥ 10)	84 (39.8)
Work productivity, % (WPAI‐GH)	
Presenteeism, *n* = 153	45.1 ± 29.5
Absenteeism, *n* = 159	13.5 ± 25.9
Overall work impairment, *n* = 153	48.9 ± 31.1
Activity impairment, *n* = 160^b^	47.7 ± 28.9
Indirect cost, 1000 yen	
Presenteeism cost, *n* = 151^c^	1564.1 ± 1077.3
Absenteeism cost, *n* = 157^c^	440.8 ± 832.8
Indirect cost, *n* = 151^c^	1688.0 ± 1115.7
QOL (HUI3)	0.461 ± 0.247
Vision	0.912 ± 0.142
Hearing	0.928 ± 0.196
Speech	0.787 ± 0.227
Ambulation	0.995 ± 0.028
Dexterity	0.993 ± 0.048
Emotion	0.637 ± 0.337
Cognition	0.640 ± 0.277
Pain	0.775 ± 0.247
Medical resource use (previous 3 months)	
Number of hospital visits for bipolar disorder	4.0 ± 3.2
Number of all hospitalizations	0.1 ± 0.5
Duration of all hospitalizations in hospitalized participants, days (*n* = 8^d^)	14.4 ± 12.7
Number of hospitalizations for bipolar disorder	0.0 ± 0.3
Duration of hospitalizations for bipolar disorder in hospitalized participants, days (*n* = 3^d^)	14.0 ± 19.9

*Note:* Data are presented as *n* (%) or mean ± standard deviation.

Abbreviations: AIS, Athens Insomnia Scale; COBRA, cognitive complaints in bipolar disorder rating assessment; ESS, epworth sleepiness scale; HUI3, health utilities index mark 3; PHQ‐9, patient health questionnaire‐9; QOL, quality of life; WPAI‐GH, work productivity and activity impairment: general health.

^a^ Excludes outliers due to input error (*n* = 1).

^b^ Excludes nonrespondents due to system malfunction (*n* = 51).

^c^ Excludes respondents who did not want to answer gender (*n* = 2).

^d^ Excludes outliers due to input error (*n* = 1).

### 3.2 Correlations Between Symptoms

**FIGURE 2:**
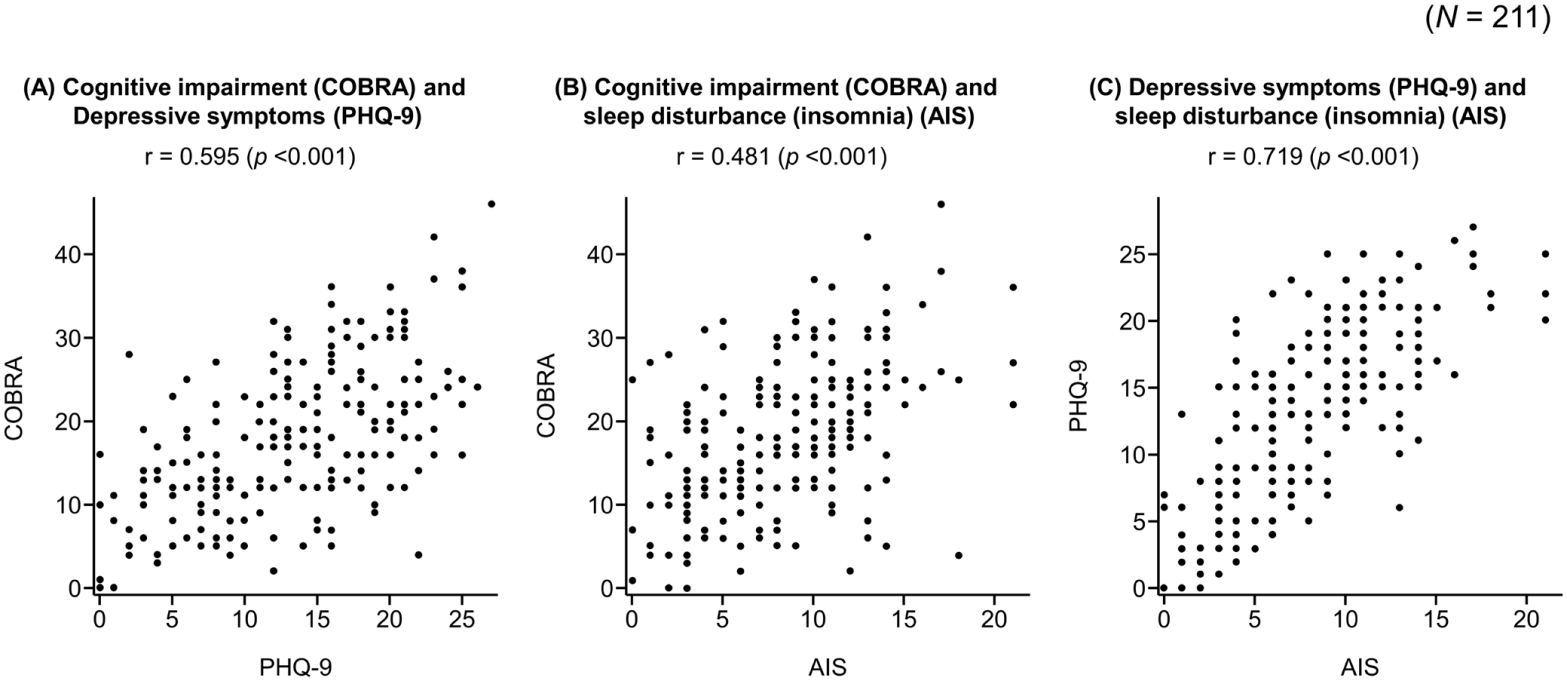
Correlations between cognitive impairment and depression (A), cognitive impairment and sleep disturbance (B), and depression and sleep disturbance (C). AIS, Athens Insomnia Scale; COBRA, Cognitive Complaints in Bipolar Disorder Rating Assessment; PHQ‐9, Patient Health Questionnaire‐9; *r*, Pearson's correlation coefficient.

Cognitive impairment (COBRA) was moderately correlated with depressive symptoms (PHQ‐9) (*r* = 0.595, *p* < 0.001) and insomnia (AIS) (*r* = 0.481, *p* < 0.001) (Figure 2). There was also a high correlation between depressive symptoms (PHQ‐9) and insomnia (AIS) (*r* = 0.719, *p* < 0.001).

### 3.3 Work Productivity Loss

**FIGURE 3:**
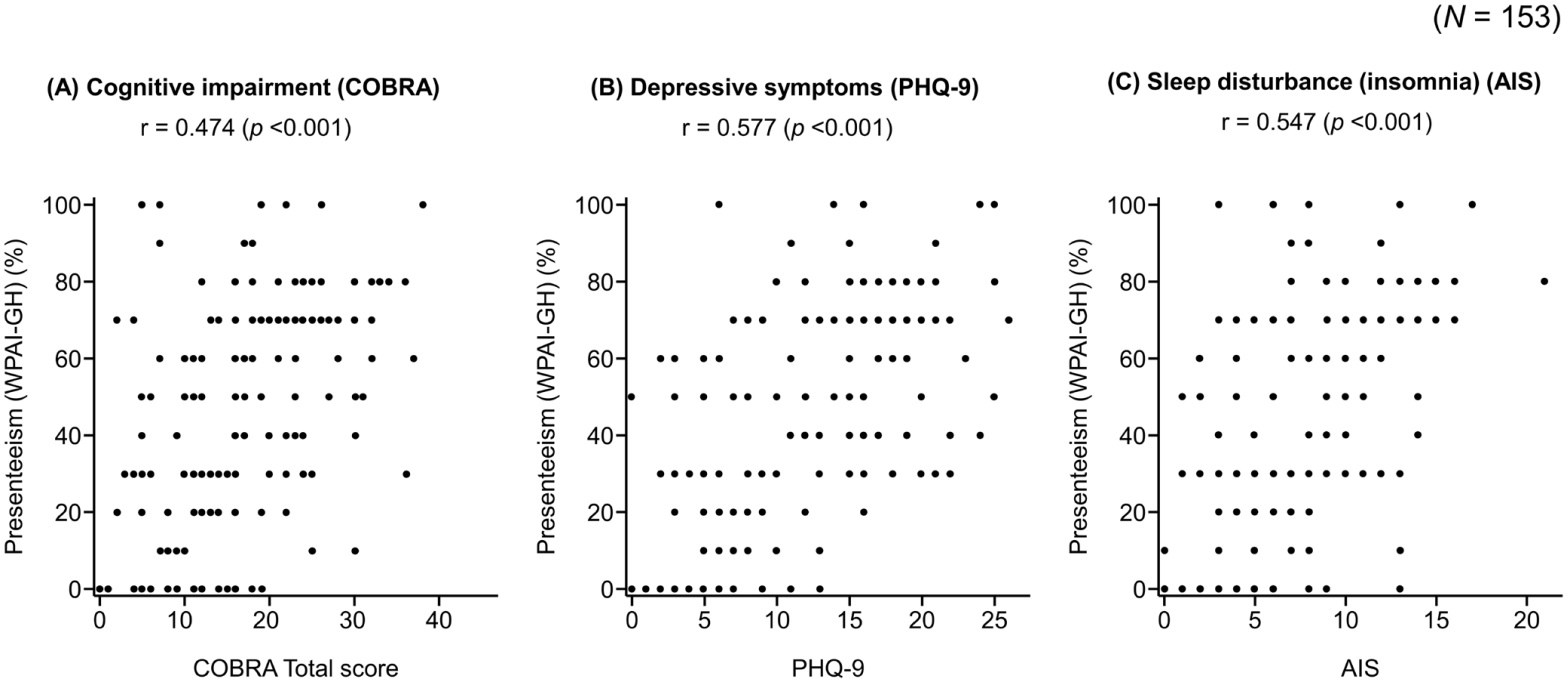
Correlations between cognitive impairment (A), depression (B), and sleep disturbance (C) with work productivity (presenteeism). AIS, Athens Insomnia Scale; COBRA, Cognitive Complaints in Bipolar Disorder Rating Assessment; PHQ‐9, Patient Health Questionnaire‐9; *r*, Pearson's correlation coefficient; WPAI‐GH, Work Productivity and Activity Impairment: General Health.

Moderate positive correlations of work productivity (presenteeism) with cognitive impairment (*r* = 0.474; *p* < 0.001), depression (*r* = 0.577, *p* < 0.001), and insomnia (*r* = 0.547, *p* < 0.001) were found (Figure 3). The results of single and multiple regression analyses for variables associated with work productivity loss are shown in Tables S1 and S2, respectively. Significant losses in work productivity (presenteeism) were found for participants with cognitive impairment, depression, somnolence, and insomnia in the single regression analysis (Table S1). Depression had the highest regression coefficient with respect to presenteeism in the single regression analysis (34.53; *p* < 0.001) (Table S1). Multiple regression analysis was performed using the explanatory variables listed in Tables 2 and S2. Depression also had the highest regression coefficient with respect to presenteeism in the multiple regression analysis (22.98, *p* < 0.001) (Table 2).

**TABLE 2:** Multiple regression analysis: Work productivity (presenteeism).

Explanatory variable	Regression coefficient	*R* ^2^ = 0.413	
		95% CI	*p*
Cognitive impairment (COBRA)			
Absence [ref]/presence	6.72	−3.06, 16.49	0.177
Depressive symptoms (PHQ‐9)			
Absence [ref]/presence	22.98	11.60, 34.35	< 0.001
Sleep disturbance (somnolence) (ESS)			
Absence [ref]/presence	0.30	−8.71, 9.31	0.947
Sleep disturbance (insomnia) (AIS)			
Absence [ref]/presence	5.97	−5.10, 17.04	0.288
Sex			
Male [ref]/female	−9.14	−17.85, −0.43	0.040
Age	−0.33	−0.85, 0.20	0.219
Duration of disease	−0.44	−1.13, 0.24	0.202
Comorbidity			
Absence [ref]/presence	2.47	−6.15, 11.08	0.572
Marital status			
Unmarried [ref]/married	−0.04	−8.94, 8.86	0.993
Smoking history			
Never smoker [ref]/current smoker	6.53	−2.88, 15.95	0.172
Never smoker [ref]/prior smoker	4.89	−8.21, 17.99	0.462
Eating habits			
Regular [ref]/irregular	5.33	−3.16, 13.82	0.217

Abbreviations: AIS, Athens Insomnia Scale; CI, confidence interval; COBRA, Cognitive Complaints in Bipolar Disorder Rating Assessment; ESS, Epworth Sleepiness Scale; PHQ‐9, Patient Health Questionnaire‐9; *R* ^2^, coefficient of determination; ref, reference.

For the population of participants without severe depressive symptoms (PHQ‐9 ≤ 19), a moderate positive correlation between work productivity loss (presenteeism) and cognitive impairment was identified (*r* = 0.412; *p* < 0.001) (Figure S1a). Single regression analysis showed that cognitive impairment and insomnia strongly affected presenteeism (22.47, *p* < 0.001 and 25.16, *p* < 0.001, respectively) (Table S3). In the multiple regression analysis, cognitive impairment and insomnia were also shown to strongly affect presenteeism (13.91, *p* = 0.007 and 13.80, *p* = 0.016, respectively) (Table S4).

For the population of participants without moderately severe or severe depressive symptoms (PHQ‐9 ≤ 14), there was a mild positive correlation between work productivity loss (presenteeism) and cognitive impairment (*r* = 0.386; *p* < 0.001) (Figure S2a). Findings from the single and multiple regression analyses for variables associated with work productivity loss among participants without moderately severe or severe depressive symptoms are shown in Tables S5 and S6, respectively. The multiple regression analysis showed that insomnia strongly affected presenteeism (23.14, *p* = 0.011) (Table S6).

### 3.4 QOL

**FIGURE 4:**
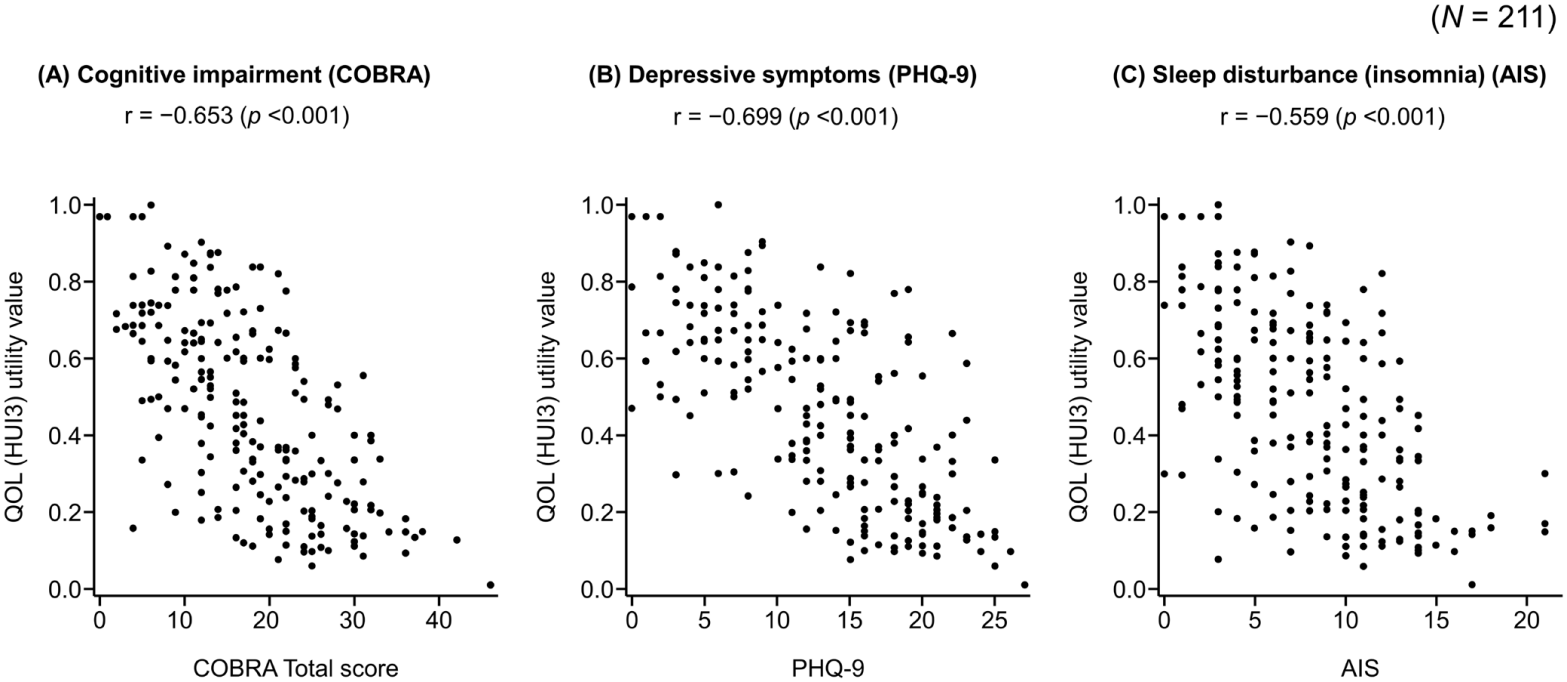
Correlations between cognitive impairment (A), depression (B), and sleep disturbance (C) with QOL. AIS, Athens Insomnia Scale; COBRA, Cognitive Complaints in Bipolar Disorder Rating Assessment; HUI3, Health Utilities Index Mark 3; PHQ‐9, Patient Health Questionnaire‐9; *r*, Pearson's correlation coefficient; QOL, quality of life.

QOL showed moderate negative correlations with cognitive impairment (*r* = −0.653, *p* < 0.001), depression (*r* = −0.699, *p* < 0.001), and insomnia (*r* = −0.559, *p* < 0.001) (Figure 4). QOL was significantly reduced in participants with cognitive impairment, depression, somnolence, and insomnia in the single regression analysis (cognitive impairment, −0.29 *p* < 0.001; depression, −0.34 *p* < 0.001; somnolence, −0.10 *p* = 0.004; insomnia, −0.28 *p* < 0.001) (Table S7). All these factors except somnolence were significantly associated with QOL reduction in the multiple regression analysis (cognitive impairment, −0.12 *p* < 0.001; depression, −0.12 *p* = 0.010; insomnia, −0.16 *p* < 0.001) (Table 3).

**TABLE 3:** Multiple regression analysis: QOL (HUI3).

*R* ^2^ = 0.593			
Explanatory variable	Regression coefficient	95% CI	*p*
Cognitive impairment (COBRA)			
Absence [ref]/presence	−0.12	−0.19, −0.05	< 0.001
Depressive symptoms (PHQ‐9)			
Absence [ref]/presence	−0.12	−0.21, −0.03	0.010
Sleep disturbance (somnolence) (ESS)			
Absence [ref]/presence	0.02	−0.05, 0.08	0.552
Sleep disturbance (insomnia) (AIS)			
Absence [ref]/presence	−0.16	−0.24, −0.08	< 0.001
Sex			
Male [ref]/female	0.09	0.03, 0.16	0.004
Age	−0.00	−0.00, 0.00	0.701
Duration of disease	−0.00	−0.01, 0.00	0.600
Comorbidity			
Absence [ref]/presence	−0.04	−0.10, 0.03	0.262
Marital status			
Unmarried [ref]/married	0.00	−0.06, 0.07	0.923
Smoking history			
Never smoker [ref]/current smoker	−0.02	−0.09, 0.05	0.605
Never smoker [ref]/prior smoker	−0.01	−0.10, 0.09	0.898
Eating habits			
Regular [ref]/irregular	−0.03	−0.09, 0.03	0.376
Work productivity (WPAI‐GH); presenteeism	−0.00	−0.00, 0.00	0.093
Work productivity (WPAI‐GH); absenteeism	−0.00	−0.00, 0.00	0.233

Abbreviations: AIS, athens insomnia scale; CI, confidence interval; COBRA, cognitive complaints in bipolar disorder rating assessment; ESS, epworth sleepiness scale; HUI3, health utilities index mark 3; PHQ‐9, patient health questionnaire‐9; QOL, quality of life; *R* ^2^, coefficient of determination; ref, reference; WPAI‐GH, work productivity and activity impairment: general health.

## 4 Discussion

This was a cross‐sectional interim analysis of baseline data from an ongoing 48‐week longitudinal study of Japanese outpatients with bipolar disorder who were being treated at a medical institution at the time of the first questionnaire assessment. The primary endpoint of the study is the change from baseline in cognitive function and work productivity at 48 weeks. The present interim analysis identified a correlation between cognitive impairment and work productivity loss at baseline, but could not identify whether cognitive impairment was an independent factor associated with work productivity loss. This may have been because bipolar patients with severe depressive symptoms (despite being employed) were included in this study. In participants who did not have severe depressive symptoms, insomnia and cognitive impairment were associated with work productivity loss. In patients who did not have moderately severe or severe depressive symptoms, insomnia, but not cognitive impairment, was associated with work productivity loss. Lower QOL was associated with depressive symptoms, cognitive impairment, and insomnia. The planned longitudinal analysis will examine the correlation between changes from baseline in cognitive function (COBRA) and work productivity (WPAI‐GH) at 48 weeks.

To our knowledge, this is the first study to evaluate the relationship between changes in cognitive function and changes in work productivity in patients with bipolar disorder undergoing treatment at an institution while working. This analysis demonstrated that depressive symptoms had a major effect on work productivity among working participants with bipolar disorder. This finding is consistent with previous reports that reduced work productivity is associated with increased depressive symptoms among patients with depression [40] and bipolar disorder [7] in National Health and Wellness Survey patient databases. A previous cross‐sectional study reported that both cognitive impairment and depressive symptoms affected work productivity in general adult workers in Japan, even though the PHQ‐9 score (4.2) in that study was relatively low [24]. Although the present analysis included employed participants with bipolar disorder, the mean PHQ‐9 score was 13.1, and 67.8% of participants had a score above the cutoff indicating the presence of depressive symptoms, suggesting that many participants had residual symptoms of depression. The planned longitudinal analysis will evaluate the relationship between each symptom and work productivity loss in a subgroup of participants with milder depressive symptoms.

Previous studies have reported that occupational skills and unemployment, but not work productivity loss, are associated with cognitive impairment and sleep disturbances in patients with bipolar disorder [17, 41]. Given the observed correlation between depressive symptoms and other symptoms such as cognitive impairment and insomnia, we performed several exploratory analyses in two subpopulations of participants without severe (PHQ‐9 ≤ 19) or without moderately severe or severe (PHQ‐9 ≤ 14) depressive symptoms. The multiple regression analysis showed that symptoms of cognitive impairment and insomnia affected work productivity in participants without severe depressive symptoms (PHQ‐9 ≤ 19). In participants without moderately severe or severe depressive symptoms (PHQ‐9 ≤ 14), symptoms of insomnia affected work productivity. This suggests that insomnia and cognitive impairment may be factors in the decline of work productivity in some patients with bipolar disorder, especially those with mild or moderate bipolar disorder. When considering the productivity of working patients with bipolar disorder, the main goal of treatment is to improve core depressive symptoms; however, it may be necessary to consider whether insomnia and cognitive impairment should also be treated.

In this analysis, 59.7% and 67.8% of participants had cognitive impairment or depression, respectively. Furthermore, the mean COBRA and PHQ‐9 scores for the total population exceeded those cutoffs (17.6 and 13.1, respectively). The severity of cognitive impairment and depressive symptoms was higher in this study than in previous studies. For example, a study assessing work productivity loss by severity of bipolar symptoms reported that, of patients who were employed, 30% had experienced a full episode of depressive symptoms [42]. Another study assessing occupational skills in working patients with bipolar disorder reported a mean baseline PHQ‐9 score of 10.6 [17]. Furthermore, patients with bipolar disorder who were in remission had COBRA scores ranging from 13.7 to 16.7 [23, 26]. Considering the correlation between depressive symptoms and cognitive impairment, it is likely that participants in this analysis had some residual symptoms of cognitive impairment and depression. Some patients have a strong desire or need to remain employed following treatment despite experiencing residual depression and cognitive dysfunction, highlighting the importance of symptom control to prevent the need for sick leave upon return to work. The association between sick leave and return‐to‐work events will be investigated in the 48‐week longitudinal analysis.

The results of this interim analysis suggest that when considering work productivity loss in patients with bipolar disorder who are working despite having residual symptoms, the most important treatment goal is to improve the core symptoms of bipolar disorder. However, it is also important to focus on other symptoms such as cognitive impairment and insomnia, as appropriate for each individual patient.

Depressive symptoms, insomnia, and cognitive impairment affected QOL in this analysis. Patients with bipolar disorder have impaired QOL, and those who have most recently experienced a depressive phase show the greatest reduction in QOL [43]. It has been reported that QOL decreases are greater with increasing severity of depressive symptoms [7, 40, 44]. Furthermore, patients with bipolar disorder who experience sleep disturbances, including insomnia, have a lower QOL than those without sleep problems [45], and insomnia and somnolence are correlated with QOL in these patients [46]. Cognitive impairment has also been reported to correlate with QOL in patients with bipolar disorder [21]. Our findings from the multiple regression analysis strongly support previous findings on the effects of depressive symptoms, insomnia, and cognitive impairment on QOL (HUI3). It is possible that the effect of each of these symptoms on QOL may be responsible for the reduced QOL that is observed in patients with bipolar disorder, who reportedly have lower QOL than the general population [7]. These findings highlight the importance of improving or controlling depressive symptoms, insomnia, and cognitive impairment to improve QOL in patients with bipolar disorder.

This study had several limitations that should be considered when interpreting the results. The eligibility criteria limited the study population, and it is possible that selection bias occurred as those who chose to participate may have been more interested in their disease than those who declined. Thus, the generalizability of these findings is limited. Because this was a cross‐sectional analysis, causal relationships could not be ascertained. However, causal relationships (cognitive impairment and work productivity loss) will be examined in the longitudinal analysis. The baseline participant background data may be confounding because of residual symptoms of cognitive impairment, depressive symptoms, and insomnia. However, the 48‐week study period permits longitudinal analysis, including subgroup analysis of participants with fewer residual symptoms and a comparison of work productivity loss between participants with and without cognitive impairment, taking into account this potential confounding. The survey items for each symptom were based on self‐administered rating scales; thus, the findings should be interpreted with caution as the symptom scores were not objectively evaluated by physicians and raters. Given the lack of a Japanese version of the self‐administered rating scale for manic symptoms, a core symptom of bipolar disorder, these symptoms could not be evaluated in this study. The possibility that manic symptoms may have affected work productivity and QOL cannot be ruled out. Finally, although ongoing pharmacotherapy (e.g., antipsychotics, benzodiazepines, mood stabilizers), subclassification of bipolar disorder (i.e., type 1 vs. 2), and comorbid neurodevelopmental disorders such as attention deficit hyperactivity disorder are potentially important confounding factors influencing cognitive impairment, these data were not available.

## 5 Conclusion

In this interim analysis, depressive symptoms, a core feature of bipolar disorder, were associated with lower work productivity at baseline in the study population, which included patients with severe depressive symptoms. In a subset of participants that excluded those with moderately severe or severe depressive symptoms, insomnia affected work productivity. In a subset of participants that excluded those with severe depressive symptoms, both insomnia and cognitive impairment affected work productivity. Depressive symptoms, cognitive impairment, and insomnia were associated with reduced QOL. Longitudinal analysis is needed to clarify in more detail whether improvements in cognitive impairment, depressive symptoms, and insomnia are associated with improvement in work productivity loss.

## Author Contributions

All authors contributed to the conception or design of this study, the interpretation of data, and the drafting of the manuscript, and have read and approved the final manuscript.

## Ethics Statement

The study was approved by the University of the Ryukyus Ethics Review Committee for Life Science and Medical Research Involving Human Subjects. This research complies with the Declaration of Helsinki and the Ethical Guidelines for Life Sciences and Medical Research Involving Human Subjects.

## Consent

All participants provided electronic informed consent.

## Conflicts of Interest

Y.T. has received lecture fees from the Takeda Pharmaceutical Co. Ltd. Sumitomo Pharma Co. Ltd. Otsuka Pharmaceutical Co. Ltd. Mochida Pharmaceutical Co. Ltd. Lundbeck Japan K.K., Viatris Inc. Nobelpharma Co. Ltd. Meiji Seika Pharma Co. Ltd. Kyowa Kirin Co. Ltd. Eisai Co. Ltd. MSD K.K., and Yoshitomiyakuhin Corp.; and research funding from Otsuka Pharmaceutical Co. Ltd. Meiji Seika Pharma Co. Ltd. MSD K.K., and Eisai Co. Ltd. outside the submitted work. A.S. had no competing interests. T.N. and H.M. are full‐time employees of Sumitomo Pharma Co. Ltd.

## Supporting information


**Table S1.** Single regression analysis: WPAI‐GH.
**Table S2.** Multiple regression analysis: WPAI‐GH.
**Table S3.** Single regression analysis in the PHQ‐9 ≤ 19 population: WPAI‐GH.
**Table S4.** Multiple regression analysis in the PHQ‐9 ≤ 19 population: WPAI‐GH.
**Table S5.** Single regression analysis in the PHQ‐9 ≤ 14 population: WPAI‐GH.
**Table S6.** Multiple regression analysis in the PHQ‐9 ≤ 14 population: WPAI‐GH.
**Table S7.** Single regression analysis: QOL (HUI3).
**Figure S1.** Correlations between cognitive impairment and work productivity: presenteeism (A), absenteeism (B), overall work impairment (C), and activity impairment (D) in participants with baseline PHQ‐9 ≤ 19.
**Figure S2.** Correlations between cognitive impairment and work productivity: presenteeism (A), absenteeism (B), overall work impairment (C), and activity impairment (D) in participants with baseline PHQ‐9 ≤ 14.

## Data Availability

The data from this study have not been made publicly available because the disclosure of individual data was not specified in the study protocol, and consent for public data sharing was not obtained from the participants.
