# Supplementary material for: Associations Between Cognitive Impairment, Depressive Symptoms, and Work Productivity Loss in Patients With Bipolar Disorder: A Cross‐Sectional Analysis
Source: Neuropsychopharmacol Rep. 2025 Mar 20;45(1):e70012. doi: 10.1002/npr2.70012 (PMC11925601; doi:10.1002/npr2.70012)
Supplement: Supplementary file 1 — Table S1. Single regression analysis: WPAI‐GH. Table S2. Multiple regression analysis: WPAI‐GH. Table S3. Single regression analysis in the PHQ‐9 ≤ 19 population: WPAI‐GH. Table S4. Multiple regression analysis in the PHQ‐9 ≤ 19 population: WPAI‐GH. Table S5. Single regression analysis in the PHQ‐9 ≤ 14 population: WPAI‐GH. Table S6. Multiple regression analysis in the PHQ‐9 ≤ 14 population: WPAI‐GH. Table S7. Single regression analysis: QOL (HUI3). Figure S1. Correlations between cognitive impairment and work productivity: presenteeism (A), absenteeism (B), overall work impairment (C), and activity impairment (D) in participants with baseline PHQ‐9 ≤ 19. Figure S2. Correlations between cognitive impairment and work productivity: presenteeism (A), absenteeism (B), overall work impairment (C), and activity impairment (D) in participants with baseline PHQ‐9 ≤ 14. [file NPR2-45-e70012-s001.docx]

**Supporting information**

**Table S1.** Single regression analysis: WPAI-GH

|  | **Presenteeism** | | | **Absenteeism** | | | **Overall work impairment** | | | **Activity impairment** | | |
| --- | --- | --- | --- | --- | --- | --- | --- | --- | --- | --- | --- | --- |
| **Explanatory variable** | **Regression coefficient** | **95% CI** | ***p* value** | **Regression coefficient** | **95% CI** | ***p* value** | **Regression coefficient** | **95% CI** | ***p* value** | **Regression coefficient** | **95% CI** | ***p* value** |
| Cognitive impairment (COBRA)  Absence [ref] / Presence | 24.44 | 15.50, 33.38 | <0.001 | 10.42 | 2.13, 18.71 | 0.014 | 26.50 | 17.07, 35.92 | <0.001 | 23.63 | 14.98, 32.27 | <0.001 |
| Depressive symptoms  (PHQ-9)  Absence [ref] / Presence | 34.53 | 26.29, 42.76 | <0.001 | 20.23 | 12.19, 28.27 | <0.001 | 40.33 | 32.08, 48.58 | <0.001 | 32.39 | 24.23, 40.55 | <0.001 |
| Sleep disturbance (somnolence) (ESS)  Absence [ref] / Presence | 14.14 | 4.48, 23.79 | 0.004 | 0.55 | −7.99, 9.09 | 0.899 | 13.29 | 2.99, 23.58 | 0.012 | 11.46 | 2.09, 20.82 | 0.017 |
| Sleep disturbance (insomnia) (AIS)  Absence [ref] / Presence | 26.88 | 17.99, 35.77 | <0.001 | 8.74 | 0.32, 17.16 | 0.042 | 27.94 | 18.47, 37.41 | <0.001 | 27.26 | 18.78, 35.73 | <0.001 |
| Sex  Male [ref] / Female | 0.44 | −9.77, 10.64 | 0.933 | 6.00 | −2.71, 14.71 | 0.176 | 4.50 | −6.30, 15.29 | 0.412 | 12.80 | 3.15, 22.45 | 0.010 |
| Comorbidity  Absence [ref] / Presence | 7.94 | −1.94, 17.81 | 0.114 | 0.78 | −7.76, 9.32 | 0.857 | 7.22 | −3.27, 17.71 | 0.176 | 9.83 | 0.44, 19.23 | 0.040 |
| Highest level of education  Did not graduate university [ref] / University graduate or higher | 3.00 | −7.00, 13.01 | 0.554 | −3.50 | −12.12, 5.12 | 0.424 | 0.90 | −9.72, 11.52 | 0.867 | −5.95 | −15.58, 3.69 | 0.224 |
| Marital status  Unmarried [ref] / Married | −6.93 | −16.82, 2.97 | 0.168 | −3.73 | −12.25, 4.79 | 0.388 | −9.91 | −20.34, 0.51 | 0.062 | −5.05 | −14.61, 4.51 | 0.299 |
| Living with partner  Living alone [ref] / Not living alone | −2.89 | −14.13, 8.36 | 0.613 | −1.30 | −10.99, 8.40 | 0.792 | −2.58 | −14.50, 9.34 | 0.670 | 0.00 | −10.70, 10.71 | 1.000 |
| Alcohol use  None [ref] / ≤once/month | −0.81 | −14.01, 12.38 | 0.903 | 0.54 | −10.86, 11.94 | 0.925 | −1.51 | −15.48, 12.47 | 0.831 | −5.47 | −17.96, 7.03 | 0.389 |
| None [ref] / 2–4 times/month | −5.59 | −18.67, 7.50 | 0.400 | 0.52 | −10.61, 11.66 | 0.926 | −7.49 | −21.35, 6.36 | 0.287 | 3.18 | −9.31, 15.68 | 0.616 |
| None [ref] / 2–3 times/week | −5.87 | −26.28, 14.54 | 0.570 | 0.94 | −16.89, 18.77 | 0.917 | −6.72 | −28.33, 14.89 | 0.540 | −8.88 | −27.97, 10.21 | 0.359 |
| None [ref] / ≥4 times/week | −9.75 | −26.71, 7.21 | 0.258 | −8.26 | −23.06, 6.55 | 0.272 | −9.98 | −27.94, 7.99 | 0.274 | −12.92 | −29.40, 3.57 | 0.124 |
| Smoking history  Never smoker [ref] / Current smoker | 13.22 | 2.43, 24.01 | 0.017 | 8.93 | −0.35, 18.20 | 0.059 | 15.63 | 4.24, 27.01 | 0.008 | 6.94 | −3.58, 17.46 | 0.194 |
| Never smoker [ref] / Prior smoker | 0.45 | −14.83, 15.72 | 0.954 | 1.27 | −12.18, 14.71 | 0.853 | 3.13 | −12.98, 19.25 | 0.701 | 0.24 | −14.96, 15.43 | 0.976 |
| Eating habits  Regular [ref] / Irregular | 14.31 | 4.71, 23.91 | 0.004 | 9.84 | 1.51, 18.18 | 0.021 | 17.81 | 7.76, 27.86 | <0.001 | 14.85 | 5.69, 24.01 | 0.002 |
| Age | −0.76 | −1.22, −0.29 | 0.002 | −0.59 | −0.99, −0.19 | 0.005 | −0.91 | −1.40, −0.42 | <0.001 | −0.87 | −1.31, −0.43 | <0.001 |
| Duration of current employment, years | −0.14 | −0.78, 0.49 | 0.653 | −0.42 | −0.97, 0.12 | 0.129 | −0.29 | −0.96, 0.38 | 0.387 | −0.56 | −1.15, 0.03 | 0.064 |
| Duration of disease, years | −1.03 | −1.72, −0.35 | 0.003 | −0.46 | −1.06, 0.15 | 0.138 | −1.05 | −1.77, −0.32 | 0.005 | −1.12 | −1.78, −0.47 | <0.001 |
| Age of diagnosis, years | −0.36 | −0.90, 0.19 | 0.200 | −0.50 | −0.97, −0.03 | 0.037 | −0.55 | −1.12, 0.03 | 0.062 | −0.42 | −0.94, 0.09 | 0.108 |
| Sleep duration (weekdays), hours/day | −1.75 | −5.14, 1.64 | 0.309 | −1.08 | −3.97, 1.82 | 0.463 | −0.80 | −4.40, 2.81 | 0.663 | −0.65 | −3.76, 2.46 | 0.680 |
| Sleep duration (weekends), hours/day | −0.03 | −2.17, 2.12 | 0.980 | −0.71 | −2.58, 1.16 | 0.453 | −0.04 | −2.31, 2.24 | 0.976 | 0.71 | −1.31, 2.73 | 0.489 |
| Exercise habits, steps/day | 0.00 | −0.00, 0.00 | 0.538 | −0.00 | −0.00, 0.00 | 0.101 | 0.00 | −0.00, 0.00 | 0.267 | 0.00 | −0.00, 0.00 | 0.321 |

Abbreviations: AIS, Athens Insomnia Scale; CI, confidence interval; COBRA, Cognitive Complaints in Bipolar Disorder Rating Assessment; ESS, Epworth Sleepiness Scale; PHQ-9, Patient Health Questionnaire-9; ref, reference; WPAI-GH, Work Productivity and Activity Impairment: General Health

**Table S2.** Multiple regression analysis: WPAI-GH

|  | **Presenteeism** | | | **Absenteeism** | | | **Overall work impairment** | | | **Activity impairment** | | |
| --- | --- | --- | --- | --- | --- | --- | --- | --- | --- | --- | --- | --- |
|  | R^2^ = 0.413 | | | R^2^ = 0.206 | | | R^2^ = 0.473 | | | R^2^ = 0.388 | | |
| **Explanatory variable** | **Regression coefficient** | **95% CI** | ***p* value** | **Regression coefficient** | **95% CI** | ***p* value** | **Regression coefficient** | **95% CI** | ***p* value** | **Regression coefficient** | **95% CI** | ***p* value** |
| Cognitive impairment (COBRA)  Absence [ref] / Presence | 6.72 | −3.06, 16.49 | 0.177 | −0.38 | −10.20, 9.44 | 0.939 | 5.90 | −3.92, 15.73 | 0.237 | 6.28 | −3.41, 15.98 | 0.202 |
| Depressive symptoms (PHQ-9)  Absence [ref] / Presence | 22.98 | 11.60, 34.35 | <0.001 | 22.30 | 10.85, 33.74 | <0.001 | 31.27 | 19.84, 42.70 | <0.001 | 19.85 | 8.82, 30.87 | <0.001 |
| Sleep disturbance (somnolence) (ESS)  Absence [ref] / Presence | 0.30 | −8.71, 9.31 | 0.947 | −5.47 | −14.45, 3.52 | 0.231 | −1.81 | −10.87, 7.24 | 0.693 | −3.06 | −11.81, 5.70 | 0.491 |
| Sleep disturbance (insomnia) (AIS)  Absence [ref] / Presence | 5.97 | −5.10, 17.04 | 0.288 | −6.17 | −16.98, 4.64 | 0.261 | 1.66 | −9.46, 12.78 | 0.768 | 8.58 | −2.05, 19.22 | 0.113 |
| Sex  Male [ref] / Female | −9.14 | −17.85, −0.43 | 0.040 | 1.84 | −6.81, 10.48 | 0.675 | −5.98 | −14.73, 2.78 | 0.179 | 3.96 | −4.56, 12.49 | 0.360 |
| Age | −0.33 | −0.85, 0.20 | 0.219 | −0.34 | −0.86, 0.18 | 0.196 | −0.38 | −0.90, 0.15 | 0.156 | −0.35 | −0.86, 0.16 | 0.172 |
| Duration of disease | −0.44 | −1.13, 0.24 | 0.202 | −0.04 | −0.73, 0.65 | 0.908 | −0.38 | −1.07, 0.31 | 0.274 | −0.46 | −1.12, 0.21 | 0.175 |
| Comorbidity  Absence [ref] / Presence | 2.47 | −6.15, 11.08 | 0.572 | −1.35 | −9.94, 7.24 | 0.757 | 1.73 | −6.93, 10.38 | 0.694 | 3.15 | −5.30, 11.59 | 0.462 |
| Marital status  Unmarried [ref] / Married | −0.04 | −8.94, 8.86 | 0.993 | 1.66 | −7.15, 10.47 | 0.711 | −1.70 | −10.64, 7.25 | 0.708 | 1.53 | −7.26, 10.32 | 0.731 |
| Smoking history  Never smoker [ref] / Current smoker | 6.53 | −2.88, 15.95 | 0.172 | 4.73 | −4.63, 14.09 | 0.320 | 7.48 | −1.98, 16.94 | 0.120 | 0.06 | −9.08, 9.20 | 0.989 |
| Never smoker [ref] / Prior smoker | 4.89 | −8.21, 17.99 | 0.462 | 0.32 | −12.94, 13.58 | 0.962 | 6.51 | −6.65, 19.67 | 0.330 | 3.47 | −9.51, 16.44 | 0.598 |
| Eating habits  Regular [ref] / Irregular | 5.33 | −3.16, 13.82 | 0.217 | 5.45 | −3.04, 13.93 | 0.206 | 7.90 | −0.63, 16.43 | 0.069 | 4.24 | −4.12, 12.60 | 0.318 |

Abbreviations: AIS, Athens Insomnia Scale; CI, confidence interval; COBRA, Cognitive Complaints in Bipolar Disorder Rating Assessment; ESS, Epworth Sleepiness Scale; PHQ-9, Patient Health Questionnaire-9; R^2^, coefficient of determination; ref, reference; WPAI-GH, Work Productivity and Activity Impairment: General Health

**Table S3.** Single regression analysis in the PHQ-9 ≤19 population: WPAI-GH

|  | **Presenteeism** | | | **Absenteeism** | | | **Overall work impairment** | | | **Activity impairment** | | |
| --- | --- | --- | --- | --- | --- | --- | --- | --- | --- | --- | --- | --- |
| **Explanatory variable** | **Regression coefficient** | **95% CI** | ***p* value** | **Regression coefficient** | **95% CI** | ***p* value** | **Regression coefficient** | **95% CI** | ***p* value** | **Regression coefficient** | **95% CI** | ***p* value** |
| Cognitive impairment (COBRA)  Absence [ref] / Presence | 22.47 | 12.70, 32.24 | <0.001 | 8.08 | −0.49, 16.65 | 0.064 | 23.90 | 13.59, 34.21 | <0.001 | 20.47 | 10.76, 30.19 | <0.001 |
| Sleep disturbance (somnolence) (ESS)  Absence [ref] / Presence | 16.50 | 6.16, 26.85 | 0.002 | −0.70 | −9.54, 8.14 | 0.876 | 14.75 | 3.68, 25.82 | 0.009 | 10.80 | 0.40, 21.21 | 0.042 |
| Sleep disturbance (insomnia) (AIS)  Absence [ref] / Presence | 25.16 | 14.89, 35.43 | <0.001 | 4.89 | −4.27, 14.04 | 0.293 | 24.98 | 13.99, 35.96 | <0.001 | 27.06 | 17.14, 36.99 | <0.001 |
| Sex  Male [ref] / Female | −1.88 | −12.72, 8.97 | 0.732 | 3.70 | −5.18, 12.57 | 0.411 | 1.67 | −9.79, 13.14 | 0.773 | 11.78 | 1.29, 22.27 | 0.028 |
| Comorbidity  Absence [ref] / Presence | 10.67 | 0.08, 21.27 | 0.048 | 0.75 | −8.09, 9.59 | 0.867 | 9.79 | −1.46, 21.03 | 0.088 | 9.29 | −1.13, 19.71 | 0.080 |
| Highest level of education  Did not graduate university [ref] / University graduate or higher | 3.40 | −7.32, 14.12 | 0.531 | −0.60 | −9.44, 8.24 | 0.893 | 2.14 | −9.21, 13.48 | 0.710 | −4.29 | −14.81, 6.23 | 0.421 |
| Marital status  Unmarried [ref] / Married | −3.78 | −14.47, 6.90 | 0.485 | −3.71 | −12.47, 5.06 | 0.404 | −7.15 | −18.40, 4.09 | 0.210 | −2.51 | −12.99, 7.97 | 0.637 |
| Living with partner  Living alone [ref] / Not living alone | −3.49 | −15.34, 8.37 | 0.562 | −1.00 | −10.76, 8.76 | 0.840 | −3.48 | −16.01, 9.06 | 0.584 | 0.80 | −10.85, 12.44 | 0.893 |
| Alcohol use  None [ref] / ≤once/month | −4.26 | −18.38, 9.87 | 0.552 | 3.00 | −8.71, 14.72 | 0.613 | −3.24 | −18.20, 11.73 | 0.669 | –6.75 | −20.38, 6.88 | 0.329 |
| None [ref] / 2–4 times/month | −9.26 | −23.38, 4.87 | 0.197 | −1.40 | −13.00, 10.21 | 0.812 | −9.82 | −24.78, 5.15 | 0.197 | 3.25 | −10.50, 17.00 | 0.641 |
| None [ref] / 2–3 times/week | −10.26 | −31.77, 11.26 | 0.347 | −2.66 | −20.72, 15.40 | 0.771 | −9.85 | −32.64, 12.94 | 0.394 | −11.19 | −32.40, 10.01 | 0.298 |
| None [ref] / ≥4 times/week | −13.08 | −31.71, 5.55 | 0.167 | −4.77 | −20.40, 10.86 | 0.547 | −10.69 | −30.43, 9.05 | 0.286 | −14.44 | −32.79, 3.91 | 0.122 |
| Smoking history  Never smoker [ref] / Current smoker | 12.38 | 0.45, 24.31 | 0.042 | 7.40 | −2.42, 17.22 | 0.138 | 15.62 | 3.09, 28.15 | 0.015 | 8.10 | −3.82, 20.01 | 0.181 |
| Never smoker [ref] / Prior smoker | 0.08 | −16.19, 16.36 | 0.992 | 0.89 | −12.80, 14.57 | 0.898 | 3.64 | −13.44, 20.73 | 0.674 | 0.02 | −16.30, 16.33 | 0.998 |
| Eating habits  Regular [ref] / Irregular | 12.08 | 1.31, 22.85 | 0.028 | 6.61 | −2.32, 15.54 | 0.146 | 15.00 | 3.70, 26.29 | 0.010 | 10.60 | 0.02, 21.18 | <0.050 |
| Age | −0.60 | −1.10, −0.10 | 0.018 | −0.47 | −0.88, −0.06 | 0.025 | −0.74 | −1.26, −0.22 | 0.006 | −0.67 | −1.16, −0.19 | 0.007 |
| Duration of current employment, years | 0.06 | −0.59, 0.71 | 0.863 | −0.29 | −0.83, 0.25 | 0.286 | −0.06 | −0.75, 0.63 | 0.860 | −0.42 | −1.04, 0.20 | 0.183 |
| Duration of disease, years | −0.91 | −1.67, −0.16 | 0.018 | −0.20 | −0.84, 0.43 | 0.527 | −0.86 | −1.66, −0.06 | 0.036 | −0.91 | −1.65, −0.17 | 0.016 |
| Age at diagnosis, years | −0.28 | −0.88, 0.32 | 0.354 | −0.54 | −1.03, −0.05 | 0.031 | −0.50 | −1.13, 0.13 | 0.116 | −0.35 | −0.92, 0.22 | 0.220 |
| Sleep duration (weekdays), hours/day | −2.76 | −6.53, 1.00 | 0.149 | −1.34 | −4.41, 1.72 | 0.387 | −1.57 | −5.58, 2.43 | 0.438 | −1.33 | −5.08, 2.41 | 0.482 |
| Sleep duration (weekends), hours/day | −0.25 | −2.63, 2.13 | 0.835 | −1.22 | −3.19, 0.74 | 0.220 | −0.20 | −2.72, 2.31 | 0.873 | 0.34 | −2.02, 2.70 | 0.775 |
| Exercise habits, steps/day | 0.00 | −0.00, 0.00 | 0.659 | 0.00 | −0.00, 0.00 | 0.249 | 0.00 | −0.00, 0.00 | 0.404 | 0.00 | −0.00, 0.00 | 0.328 |

Abbreviations: AIS, Athens Insomnia Scale; CI, confidence interval; COBRA, Cognitive Complaints in Bipolar Disorder Rating Assessment; ESS, Epworth Sleepiness Scale; PHQ-9, Patient Health Questionnaire-9; ref, reference; WPAI-GH, Work Productivity and Activity Impairment: General Health

**Table S4.** Multiple regression analysis in the PHQ-9 ≤19 population: WPAI-GH

|  | **Presenteeism** | | | **Absenteeism** | | | **Overall work impairment** | | | **Activity impairment** | | |
| --- | --- | --- | --- | --- | --- | --- | --- | --- | --- | --- | --- | --- |
|  | R^2^ = 0.286 | | | R^2^ = 0.113 | | | R^2^ = 0.300 | | | R^2^ = 0.272 | | |
| **Explanatory variable** | **Regression coefficient** | **95% CI** | ***p* value** | **Regression coefficient** | **95% CI** | ***p* value** | **Regression coefficient** | **95% CI** | ***p* value** | **Regression coefficient** | **95% CI** | ***p* value** |
| Cognitive impairment (COBRA)  Absence [ref] / Presence | 13.91 | 3.93, 23.89 | 0.007 | 3.20 | −6.05, 12.46 | 0.495 | 15.28 | 4.85, 25.70 | 0.004 | 11.19 | 1.34, 21.05 | 0.026 |
| Sleep disturbance (somnolence) (ESS)  Absence [ref] / Presence | 4.23 | −5.58, 14.03 | 0.395 | −6.38 | −15.56, 2.81 | 0.172 | 1.95 | −8.29, 12.19 | 0.707 | −4.63 | −14.37, 5.11 | 0.349 |
| Sleep disturbance (insomnia) (AIS)  Absence [ref] / Presence | 13.80 | 2.58, 25.02 | 0.016 | 7.25 | −2.79, 17.29 | 0.155 | 13.96 | 2.24, 25.68 | 0.020 | 20.81 | 9.64, 31.98 | <0.001 |
| Age | −0.24 | −0.77, 0.30 | 0.379 | −0.58 | −1.08, −0.08 | 0.024 | −0.46 | −1.02, 0.10 | 0.106 | −0.36 | −0.88, 0.16 | 0.173 |
| Duration of disease, years | −0.46 | −1.25, 0.33 | 0.247 | 0.38 | −0.36, 1.12 | 0.309 | −0.23 | −1.06, 0.59 | 0.576 | −0.44 | −1.20, 0.32 | 0.251 |
| Comorbidity  Absence [ref] / Presence | 2.40 | −7.25, 12.05 | 0.623 | −1.01 | −10.01, 7.99 | 0.825 | 1.91 | −8.16, 11.99 | 0.708 | 1.72 | −7.82, 11.25 | 0.722 |
| Smoking history  Never smoker [ref] / Current smoker | 9.19 | −1.48, 19.85 | 0.091 | 9.33 | −0.42, 19.08 | 0.060 | 12.50 | 1.36, 23.63 | 0.028 | 4.13 | −6.42, 14.68 | 0.440 |
| Never smoker [ref] / Prior smoker | 7.65 | −6.66, 21.96 | 0.292 | 6.31 | −7.27, 19.89 | 0.360 | 12.79 | −2.15, 27.74 | 0.093 | 6.81 | −7.42, 21.03 | 0.345 |
| Eating habits  Regular [ref] / Irregular | 5.54 | −3.91, 15.00 | 0.248 | 4.06 | −4.79, 12.91 | 0.366 | 8.37 | −1.51, 18.24 | 0.096 | 3.57 | −5.75, 12.90 | 0.450 |

Abbreviations: AIS, Athens Insomnia Scale; CI, confidence interval; COBRA, Cognitive Complaints in Bipolar Disorder Rating Assessment; ESS, Epworth Sleepiness Scale; PHQ-9, Patient Health Questionnaire-9; R^2^, coefficient of determination; ref, reference; WPAI-GH, Work Productivity and Activity Impairment: General Health

**Table S5.** Single regression analysis in the PHQ-9 ≤14 population: WPAI-GH

|  | **Presenteeism** | | | **Absenteeism** | | | **Overall work impairment** | | | **Activity impairment** | | |
| --- | --- | --- | --- | --- | --- | --- | --- | --- | --- | --- | --- | --- |
| **Explanatory variable** | **Regression coefficient** | **95% CI** | ***p* value** | **Regression coefficient** | **95% CI** | ***p* value** | **Regression coefficient** | **95% CI** | ***p* value** | **Regression coefficient** | **95% CI** | ***p* value** |
| Cognitive impairment (COBRA)  Absence [ref] / Presence | 17.98 | 5.15, 30.80 | 0.007 | 4.27 | −5.05, 13.58 | 0.365 | 19.84 | 6.51, 33.18 | 0.004 | 13.65 | 1.13, 26.17 | 0.033 |
| Depressive symptoms (PHQ-9)  Absence [ref] / Presence | 28.15 | 16.13, 40.17 | <0.001 | 19.31 | 10.95, 27.66 | <0.001 | 34.88 | 23.01, 46.75 | <0.001 | 21.61 | 9.52, 33.70 | <0.001 |
| Sleep disturbance (somnolence) (ESS)  Absence [ref] / Presence | 14.48 | 1.06, 27.91 | 0.035 | −2.57 | −12.26, 7.12 | 0.600 | 13.70 | −0.41, 27.81 | 0.057 | 7.89 | −5.24, 21.02 | 0.235 |
| Sleep disturbance (insomnia) (AIS)  Absence [ref] / Presence | 30.43 | 13.52, 47.35 | <0.001 | 12.98 | 0.88, 25.08 | 0.036 | 31.31 | 13.59, 49.04 | <0.001 | 22.36 | 5.57, 39.15 | 0.010 |
| Sex  Male [ref] / Female | −1.83 | −14.74, 11.08 | 0.779 | 3.08 | −5.92, 12.08 | 0.498 | 1.49 | −12.01, 14.99 | 0.827 | 9.24 | −2.97, 21.44 | 0.136 |
| Comorbidity  Absence [ref] / Presence | 7.38 | −5.95, 20.70 | 0.274 | −0.31 | −9.74, 9.13 | 0.949 | 8.23 | −5.69, 22.15 | 0.243 | 4.70 | −8.14, 17.53 | 0.469 |
| Highest level of education  Did not graduate university [ref] / University graduate or higher | 0.20 | −12.68, 13.08 | 0.976 | 1.43 | −7.56, 10.42 | 0.752 | −1.49 | −14.95, 11.97 | 0.826 | −5.21 | −17.45, 7.04 | 0.400 |
| Marital status  Unmarried [ref] / Married | −3.63 | −16.42, 9.15 | 0.573 | −4.20 | −13.10, 4.71 | 0.351 | −6.78 | −20.09, 6.54 | 0.314 | 0.16 | −12.10, 12.42 | 0.980 |
| Living with partner  Living alone [ref] / Not living alone | −5.02 | −19.48, 9.45 | 0.492 | −10.33 | −20.14,  −0.51 | 0.039 | −6.52 | −21.61, 8.58 | 0.393 | 0.72 | −13.21, 14.64 | 0.919 |
| Alcohol use  None [ref] / ≤once/month | −10.65 | −28.01, 6.70 | 0.225 | −0.67 | −13.04, 11.70 | 0.915 | −9.17 | −27.46, 9.11 | 0.321 | −16.30 | −32.37,  −0.23 | 0.047 |
| None [ref] / 2–4 times/month | −10.68 | −27.79, 6.44 | 0.218 | 2.13 | −9.91, 14.17 | 0.725 | −8.63 | −26.66, 9.41 | 0.344 | −0.59 | −16.66, 15.48 | 0.942 |
| None [ref] / 2–3 times/week | −12.82 | −39.24, 13.60 | 0.337 | −3.81 | −22.73, 15.11 | 0.690 | −12.95 | −40.79, 14.90 | 0.357 | −14.87 | −39.68, 9.93 | 0.236 |
| None [ref] / ≥4 times/week | −15.15 | −36.86, 6.55 | 0.1686 | −0.80 | −16.31, 14.72 | 0.919 | −9.58 | −32.46, 13.30 | 0.407 | −17.54 | −37.92, 2.84 | 0.091 |
| Smoking history  Never smoker [ref] / Current smoker | 16.01 | 0.61, 31.41 | 0.042 | 9.86 | −0.85, 20.56 | 0.071 | 21.96 | 6.23, 37.69 | 0.007 | 6.85 | −8.22, 21.93 | 0.368 |
| Never smoker [ref] / Prior smoker | 7.51 | −9.76, 24.79 | 0.389 | 6.81 | −5.43, 19.05 | 0.272 | 13.34 | −4.31, 30.99 | 0.136 | 6.29 | −10.63, 23.21 | 0.462 |
| Eating habits  Regular [ref] / Irregular | 12.59 | −1.25, 26.43 | 0.074 | −2.79 | −12.71, 7.13 | 0.577 | 13.84 | −0.61, 28.28 | 0.060 | 9.33 | −4.06, 22.73 | 0.170 |
| Age | −0.22 | −0.87, 0.43 | 0.503 | −0.21 | −0.66, 0.25 | 0.363 | −0.34 | −1.02, 0.34 | 0.326 | −0.20 | −0.82, 0.42 | 0.525 |
| Duration of current employment, years | 0.22 | −0.50, 0.95 | 0.540 | −0.14 | −0.65, 0.37 | 0.588 | 0.15 | −0.61, 0.91 | 0.693 | −0.13 | −0.82, 0.56 | 0.711 |
| Duration of disease, years | −0.82 | −1.72, 0.08 | 0.075 | −0.15 | −0.79, 0.49 | 0.633 | −0.74 | −1.68, 0.21 | 0.126 | −0.73 | −1.59, 0.14 | 0.098 |
| Age at diagnosis, years | 0.25 | −0.49, 0.98 | 0.506 | −0.17 | −0.69, 0.35 | 0.516 | 0.05 | −0.73, 0.82 | 0.908 | 0.20 | −0.49, 0.89 | 0.562 |
| Sleep duration (weekdays), hours/day | −2.93 | −7.97, 2.12 | 0.252 | 1.83 | −1.73, 5.40 | 0.309 | −1.28 | −6.59, 4.03 | 0.633 | −2.01 | −6.85, 2.84 | 0.412 |
| Sleep duration (weekends), hours/day | −0.90 | −4.54, 2.75 | 0.626 | 0.13 | −2.45, 2.71 | 0.920 | −0.44 | −4.26, 3.38 | 0.819 | −0.00 | −3.51, 3.51 | 0.998 |
| Exercise habits, steps/day | 0.00 | −0.00, 0.00 | 0.900 | 0.00 | −0.00, 0.00 | 0.235 | 0.00 | −0.00, 0.00 | 0.792 | 0.00 | −0.00, 0.00 | 0.511 |

Abbreviations: AIS, Athens Insomnia Scale; CI, confidence interval; COBRA, Cognitive Complaints in Bipolar Disorder Rating Assessment; ESS, Epworth Sleepiness Scale; HUI3, Health Utilities Index Mark 3; PHQ-9, Patient Health Questionnaire-9; ref, reference; WPAI-GH, Work Productivity and Activity Impairment: General Health; QOL, quality of life

**Table S6.** Multiple regression analysis in the PHQ-9 ≤14 population: WPAI-GH

|  | **Presenteeism** | | | **Absenteeism** | | | **Overall work impairment** | | | **Activity impairment** | | |
| --- | --- | --- | --- | --- | --- | --- | --- | --- | --- | --- | --- | --- |
| **Explanatory variable** | **Regression coefficient** | **95% CI** | ***p* value** | **Regression coefficient** | **95% CI** | ***p* value** | **Regression coefficient** | **95% CI** | ***p* value** | **Regression coefficient** | **95% CI** | ***p* value** |
| Cognitive impairment (COBRA)  Absence [ref] / Presence | 8.09 | −5.36, 21.53 | 0.235 | 2.70 | −7.56, 12.95 | 0.602 | 9.69 | −3.96, 23.33 | 0.161 | 8.36 | −5.51, 22.24 | 0.234 |
| Sleep disturbance (somnolence) (ESS)  Absence [ref] / Presence | 5.76 | −8.37, 19.90 | 0.419 | −5.67 | −16.43, 5.08 | 0.297 | 5.35 | −9.00, 19.69 | 0.460 | −1.32 | −15.90, 13.26 | 0.857 |
| Sleep disturbance (insomnia) (AIS)  Absence [ref] / Presence | 23.14 | 5.49, 40.79 | 0.011 | 14.07 | 1.07, 27.07 | 0.034 | 23.87 | 5.96, 41.79 | 0.010 | 18.53 | 0.31, 36.76 | 0.046 |
| Smoking history  Never smoker [ref] / Current smoker | 13.00 | −1.68, 27.67 | 0.082 | 7.79 | −3.38, 18.97 | 0.169 | 18.43 | 3.54, 33.32 | 0.016 | 2.96 | −12.16, 18.08 | 0.697 |
| Never smoker [ref] / Prior smoker | 13.08 | −3.10, 29.26 | 0.111 | 7.33 | −5.15, 19.81 | 0.246 | 19.56 | 3.15, 35.98 | 0.020 | 10.25 | −6.40, 26.89 | 0.224 |
| Eating habits  Regular [ref] / Irregular | 10.76 | −2.36, 23.88 | 0.107 | −2.40 | −12.46, 7.67 | 0.637 | 12.66 | −0.66, 25.97 | 0.062 | 8.28 | −5.24, 21.80 | 0.226 |
| Duration of disease, years | −0.32 | −1.19, 0.55 | 0.462 | −0.09 | −0.75, 0.57 | 0.788 | −0.21 | −1.09, 0.68 | 0.644 | −0.41 | −1.31, 0.48 | 0.362 |

Abbreviations: AIS, Athens Insomnia Scale; CI, confidence interval; COBRA, Cognitive Complaints in Bipolar Disorder Rating Assessment; ESS, Epworth Sleepiness Scale; PHQ-9, Patient Health Questionnaire-9; ref, reference; WPAI-GH, Work Productivity and Activity Impairment: General Health

**Table S7.** Single regression analysis: QOL (HUI3)

| **Explanatory variable** | **Regression coefficient** | **95% CI** | ***p* value** |
| --- | --- | --- | --- |
| Cognitive impairment (COBRA)  Absence [ref] / Presence | −0.29 | −0.35, −0.23 | <0.001 |
| Depressive symptoms (PHQ-9)  Absence [ref] / Presence | −0.34 | −0.40, −0.28 | <0.001 |
| Sleep disturbance (somnolence) (ESS)  Absence [ref] / Presence | −0.10 | −0.17, −0.04 | 0.004 |
| Sleep disturbance (insomnia) (AIS)  Absence [ref] / Presence | −0.28 | −0.34, −0.22 | <0.001 |
| Sex  Male [ref] / Female | 0.00 | −0.07, 0.07 | 0.994 |
| Comorbidity  Absence [ref] / Presence | −0.09 | −0.16, −0.02 | 0.015 |
| Highest level of education  Did not graduate university [ref]] / University graduate or higher | 0.08 | 0.01, 0.15 | 0.035 |
| Marital status  Unmarried [ref] / Married | 0.05 | −0.02, 0.12 | 0.150 |
| Living with partner  Living alone [ref] / Not living alone | 0.06 | −0.02, 0.13 | 0.169 |
| Alcohol use  None [ref] / ≤once/month | 0.01 | −0.08, 0.11 | 0.795 |
| None [ref] / 2–4 times/month | 0.04 | −0.06, 0.14 | 0.407 |
| None [ref] / 2–3 times/week | 0.11 | −0.04, 0.25 | 0.153 |
| None [ref] / ≥4 times/week | 0.08 | −0.04, 0.19 | 0.211 |
| Smoking history  Never smoker [ref] / Current smoker | −0.13 | −0.21, −0.05 | <0.001 |
| Never smoker [ref] / Prior smoker | 0.01 | −0.10, 0.12 | 0.860 |
| Eating habits  Regular [ref] / Irregular | −0.14 | −0.21, −0.07 | <0.001 |
| Age | 0.00 | −0.00, 0.01 | 0.320 |
| Duration of current employment, years | 0.00 | −0.00, 0.01 | 0.274 |
| Duration of disease, years | 0.00 | −0.00, 0.01 | 0.441 |
| Age at diagnosis, years | 0.00 | −0.00, 0.01 | 0.588 |
| Sleep duration (weekdays), hours/day | −0.00 | −0.03, 0.02 | 0.726 |
| Sleep duration (weekends), hours/day | −0.01 | −0.02, 0.01 | 0.356 |
| Exercise habits, steps/day | 0.00 | 0.00, 0.00 | 0.563 |
| Work productivity (WPAI-GH) |  |  |  |
| Presenteeism | −0.00 | −0.01, −0.00 | <0.001 |
| Absenteeism | −0.00 | −0.01, −0.00 | <0.001 |
| Overall work impairment | −0.00 | −0.01, −0.00 | <0.001 |
| Activity impairment | −0.00 | −0.01, −0.00 | <0.001 |
| Indirect cost, 1000 yen |  |  |  |
| Presenteeism cost | 0.00 | 0.00, 0.00 | <0.001 |
| Absenteeism cost | 0.00 | 0.00, 0.00 | <0.001 |
| Indirect cost | 0.00 | 0.00, 0.00 | <0.001 |

Abbreviations: AIS, Athens Insomnia Scale; CI, confidence interval; COBRA, Cognitive Complaints in Bipolar Disorder Rating Assessment; ESS, Epworth Sleepiness Scale; HUI3, Health Utilities Index Mark 3; PHQ-9, Patient Health Questionnaire-9; QOL, quality of life; ref, reference; WPAI-GH, Work Productivity and Activity Impairment: General Health

**
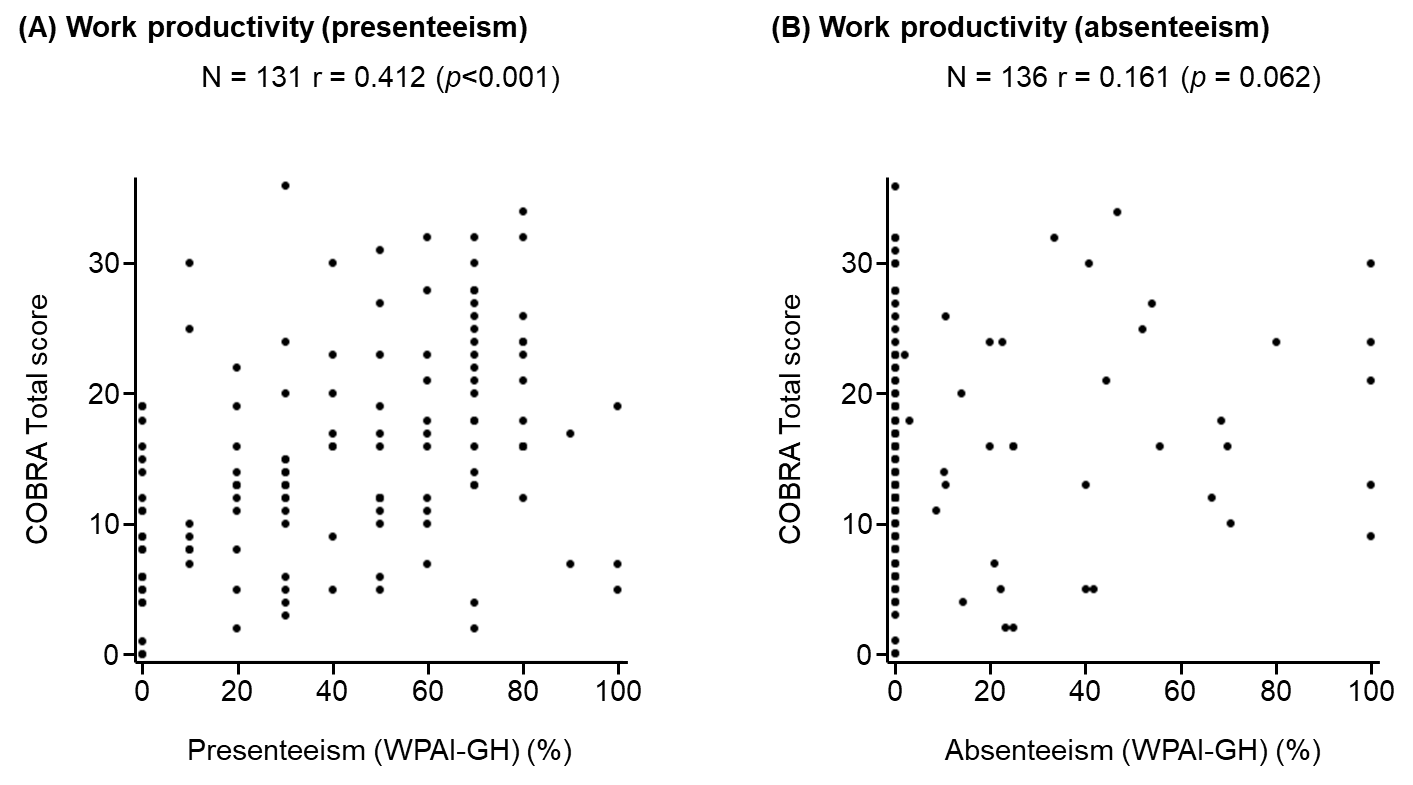
**

**
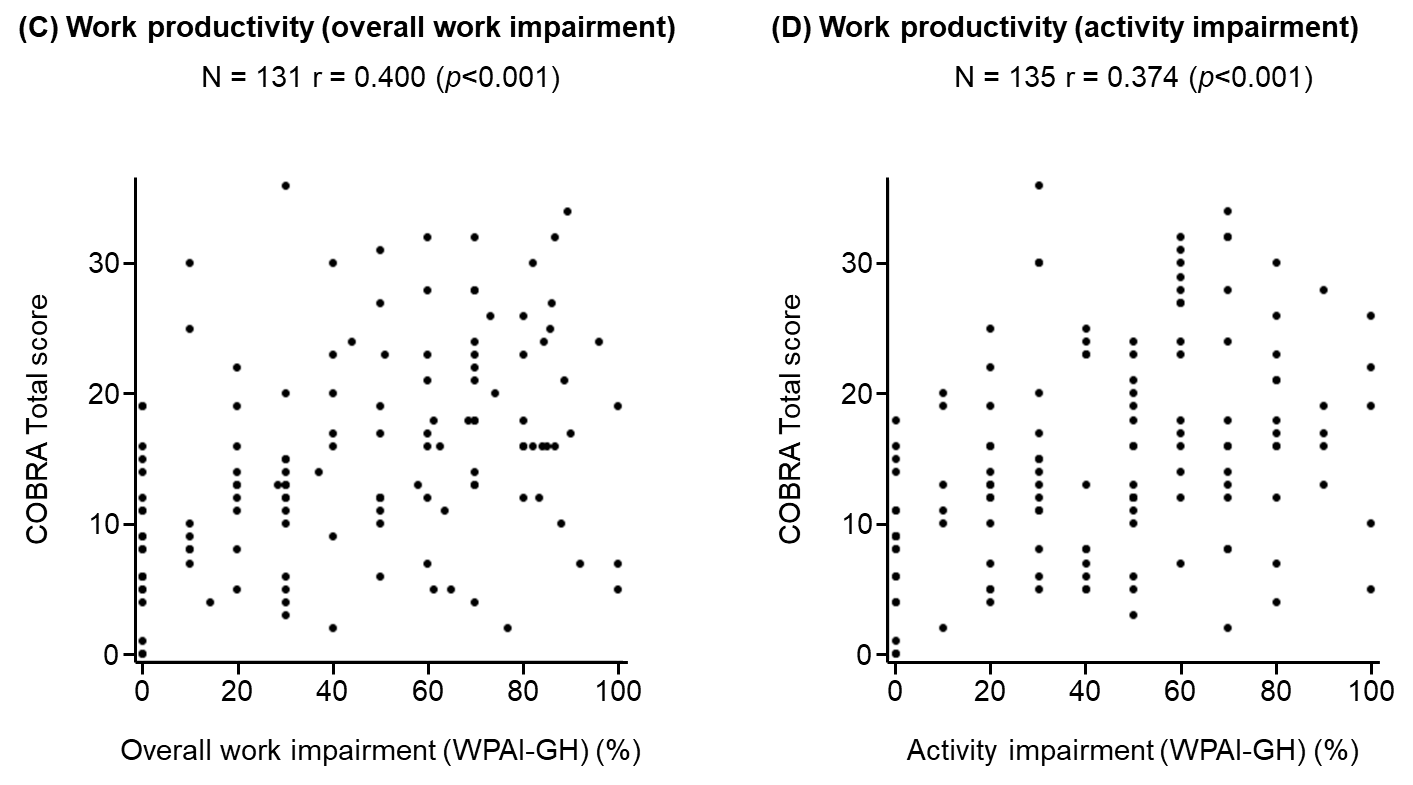
**

**Figure S1.** Correlations between cognitive impairment and work productivity: presenteeism (A), absenteeism (B), overall work impairment (C), and activity impairment (D) in participants with baseline PHQ-9 ≤19.

Abbreviations: COBRA, Cognitive Complaints in Bipolar Disorder Rating Assessment; PHQ-9, Patient Health Questionnaire-9; r, Pearson correlation coefficient; WPAI-GH, Work Productivity and Activity Impairment: General Health

**
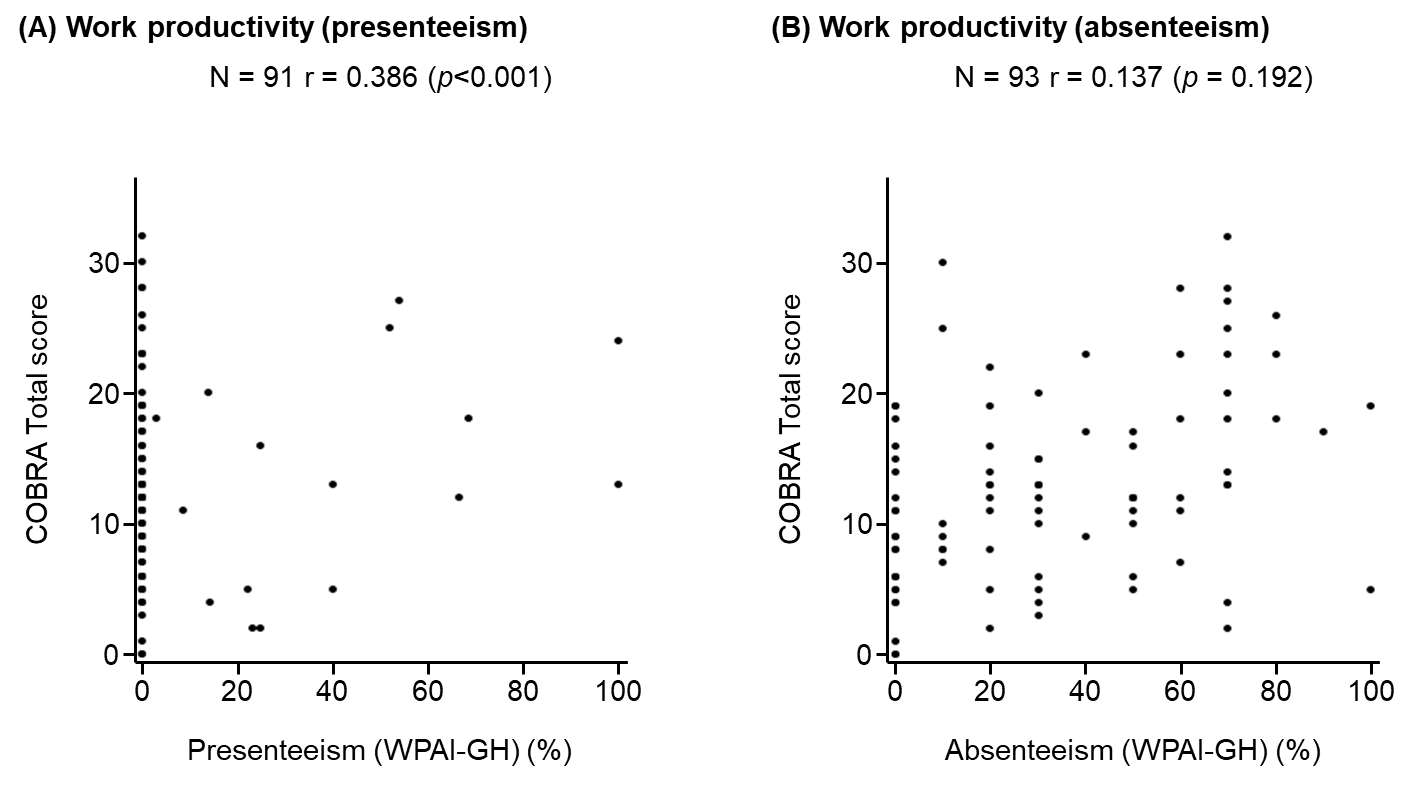
**

**
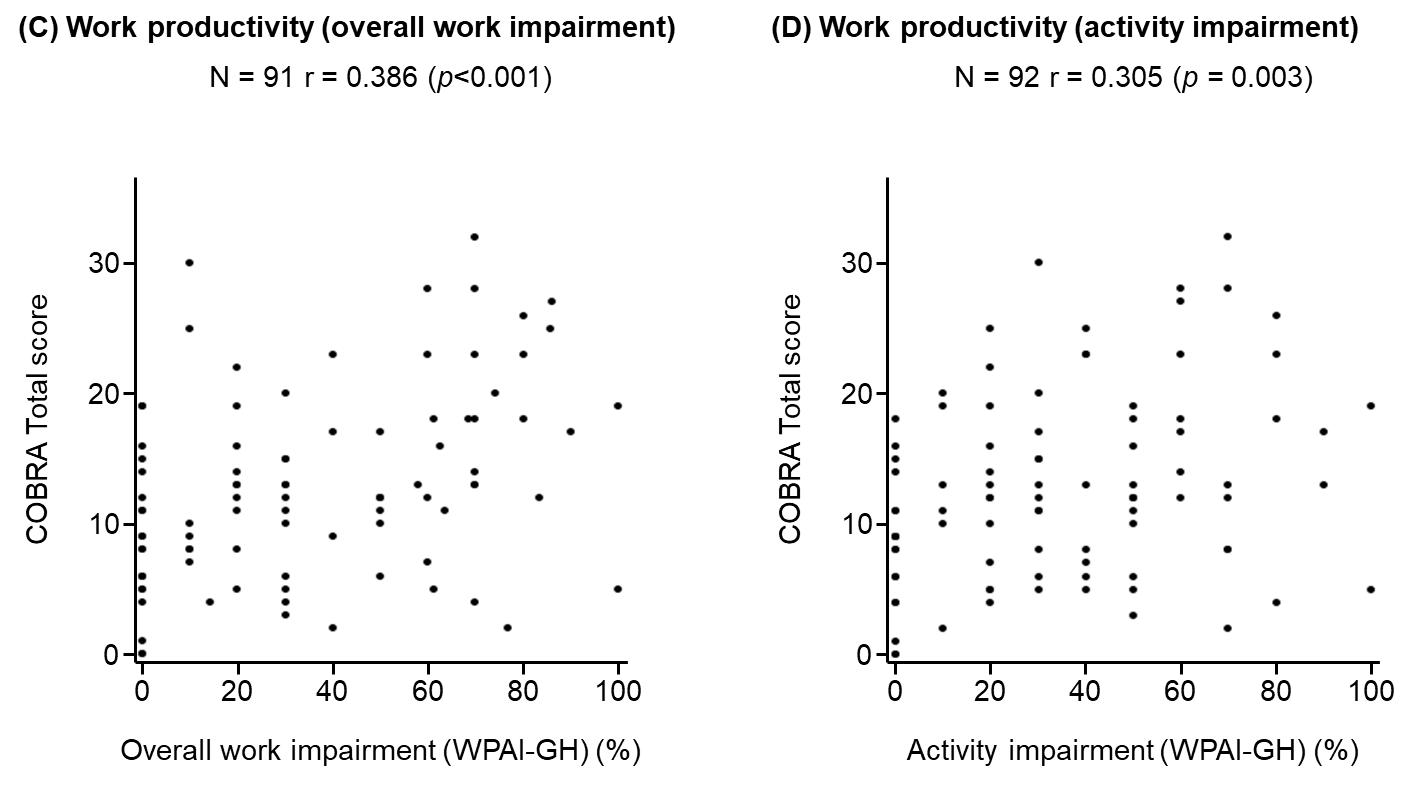
**

**Figure S2.** Correlations between cognitive impairment and work productivity: presenteeism (A), absenteeism (B), overall work impairment (C), and activity impairment (D) in participants with baseline PHQ-9 ≤14.

Abbreviations: COBRA, Cognitive Complaints in Bipolar Disorder Rating Assessment; PHQ-9, Patient Health Questionnaire-9; r, Pearson correlation coefficient; WPAI-GH, Work Productivity and Activity Impairment: General Health
